# Treating the primary in low burden metastatic prostate cancer: Where do we stand?

**DOI:** 10.1097/MD.0000000000023715

**Published:** 2020-12-18

**Authors:** Hua-Chun Luo, Zhi-Chao Fu, Xin-Peng Wang, Lv-Juan Cai, Feng-Mei Wang, Qin Yin, Guishan Lin, Zhong-Hua Chen, Shao-Guang Liao

**Affiliations:** a Department of Radiation Oncology; b Department of Gynaecology and Obstetrics, The 900th Hospital of Joint Logistics Force (Xiamen Dongfang Hospital), Fuzhou; c Department of Oncology, Longyan People's Hospital, Longyan; d Department of Radiation Oncology, Fujian Province's Hospital, Fujian; e Department of Oncology, Taizhou First People's Hospital (Huangyan Hospital, Wenzhou Medical University), Taizhou, Zhejiang, China.

**Keywords:** acute radiation injury, efficacy, endocrine therapy, low burden metastatic prostate cancer, quality of life

## Abstract

On the basis of endocrine therapy for patients with low burden metastatic prostate cancer (LBMP), the clinical efficacy and quality of life were compared between prostate-only directed radiotherapy (PODT) and prostate and metastasis radiotherapy (PMRT).

From November 2009 to November 2015, total 91 patients newly diagnosed with LBMP were retrospectively analyzed, of which 52 patients received PODT and 39 patients received PMRT. The biochemical failure free interval (IBF), prostate specific survival (PCSS), and overall survival (OS) time were compared between the 2 groups, and expanded prostate cancer index composite (EPIC) scale was used to evaluate the difference in quality of life between the 2 groups.

The median IBF of the PODT group was 31 months, which was significantly lower than the 39 months of the PMRT group (*P* < .05); the 5-year OS and PCSS were 58.9%, 65.3% in PODT group, and 58.9%, 71.79% in PMRT group, respectively. There was no significant between the 2 groups (*P* > .05); the side effects of acute radiotherapy in PMRT group were significantly higher than PODT group (*P* < .05), especially in bone marrow suppression and gastrointestinal reactions; The scores of urinary system function and intestinal system function in PMRT group were significantly higher than PODT group at the end of radiotherapy, 3 months after radiotherapy, and 6 months after radiotherapy (*P* < .05). The score of sexual function in PMRT group was significantly lower than that in PODT group after radiotherapy (*P* < .05), and higher than that in PORT group at other follow-up time points (*P* < .05). The hormone function was decreased at each follow-up time point in 2 groups, and there was no significant difference between the 2 groups (*P* > .05).

Patients with LBMP receiving PMRT can improve IBF, but cannot increase PCSS and OS, and increase the incidence of acute radiation injury.

## 1 Introduction

Prostate cancer is one of the common malignant tumors. About 70% of patients have distant metastases at the time of diagnosis in most developing countries.^[1]^ With the progress of surgery, radiotherapy and endocrine therapy, the prognosis of patients with distant metastasis of prostate cancer is significantly improved, but the treatment of low burden metastatic prostate cancer (LBMP) remains controversial.^[2]^ Several clinical trials have been conducted to investigate the impact of local prostate treatment on prognosis. A prospective randomized controlled study has confirmed that combined prostate-only directed radiotherapy (PODT) can improve the overall survival compared with endocrine therapy alone.^[3]^ However, there are still many defects need to explain, such as quality of life, the scope of radiotherapy, target volume, etc, especially whether the prostate and metastasis radiotherapy (PAMT) can improve the prognosis, these key indicators affect the choice of treatment for patients with LBMP. This study used a retrospective cohort study to compare the differences in prognosis and quality of life with 2 radiation therapy methods, PODT, and prostate and metastasis radiotherapy (PMRT).

## 2 Materials and methods

### 2.1 General information

The clinical data of newly diagnosed LBMP patients in our hospital from November 2009 to November 2015 were selected. Inclusion criteria

(1) Histopathologically confirmed prostate acinar cell carcinoma;
(2) whole body imaging and whole spinal magnetic resonance imaging confirmed that there are ≤4 metastases;
(3) patients had no visceral metastasis by chest CT and whole abdomen enhanced CT.
(4) ECOG score 0 to 2 points;
(5) age ≤ 75 years;
(6) receive radiotherapy for prostate lesions and/or metastases;
(7) receive standard continuous androgen blockade endocrine therapy for more than 2 years;
(8) complete clinical data.

Exclusion criteria:

(1) have previously received prostate cancer treatment (including surgery, radiotherapy, endocrine therapy, chemotherapy, or other);
(2) combined with uncontrollable underlying diseases;
(3) Previous history of malignant tumors;
(4) patients who do not want to be followed up for a long time;
(5) Patients with mental system diseases;
(6) The radiotherapy course has not been completed.

This study was approved by the hospital ethics committee, and all patients signed informed consent form.

### 2.2 Treatment methods

#### 2.2.1 Endocrine therapy

All subjects received standard androgen-blocking endocrine therapy: Bicalutamide (AstraZeneca), 5 mg once daily, Goserelin acetate (AstraZeneca) 3.6 mg subcutaneously, once/28 days.

#### 2.2.2 Radiotherapy

All subjects received radiation therapy. Positioning method: empty the bladder and rectum before positioning, drink 600 ml of water, hold urine for 30 min, supine position, fix with thermoplastic film combined with vacuum pad. Scanning with Philips 4.0 large-aperture analog CT positioning machine, the scanning range is from the upper edge of the 5th lumbar vertebra to 2 cm below the anus (if there is a vertebral metastasis, the upper boundary is 5 cm from the upper edge of the vertebral metastasis), and the scanning layer thickness is 3 mm. The scanned images were transmitted from the CT simulation workstation to the Varian Eclipse TPS, and the target area, organ at risk (OAR), and normal organs were delineated. The clinical target volume (CTV) of the prostate includes the prostate (with or without calcification) visible on the magnetic resonance T2 image and bilateral seminal vesicles (2–2.5 cm outside the prostate lesion), and 1 cm outside the CTV head and foot. Putting 0.3 cm outside the back boundary and 0.5 cm outside the other direction constitutes a PTV. The prescription dose is 2.5 Gy/time for a total of 33 times and the total dose is 72.5 Gy. GTV shows pelvic metastatic lymph nodes (shorter diameter at the largest level of the tumor is greater than 1 cm) and/or vertebral metastases on MR. 0.5 cm outside is PGTV. The 95% PGTV dose is 55 Gy/25 f.

The radiotherapy target area of PODT is PTV; the radiotherapy target area of PAMT is PTV + PGTV. The delineation of normal tissue and OAR is in accordance with the RTOG standard, requiring 95% of the PTV volume to receive more than 100% of the prescribed dose. OAR dose limits are: 25% rectal volume ≤70 Gy, 30% bladder volume ≤65 Gy, and 5% bilateral femoral head volume ≤50 Gy.

### 2.3 Observation indicators

2.3

#### 2.3.1 Survival time

The interval to biochemical failure (IBF), prostate cancer specific survival (PCSS), and overall survival (OS) of all patients were observed. IBF is defined as: the time from prostate cancer diagnosis to biochemical recurrence of PSA, in which biochemical recurrence is increased by ≥2 ng/ml based on the lowest value of PSA; PCSS is defined as the time from prostate cancer diagnosis to complications due to prostate cancer or prostate progression leading to death of the patient. The overall survival time is defined as the time from the diagnosis of prostate cancer to the death of the patient for any reason.

#### 2.3.2 Quality of life evaluation

An expanded prostate cancer index composite (EPIC) was used to evaluate the quality of life during follow-up. The scale could evaluate the function in 4 fields, including urinary system function, intestinal function, sexual function, and hormonal function. Questionnaire points are in the form of a percentage system with a score range of 0 to 100 points. The higher scores in EPIC, the better quality of life.

The time follow-up points of the questionnaire survey were the first day of radiotherapy (time point A), the day of radiotherapy end (time point B), 3 months after the end of radiotherapy (time point C), and 6 months after the end of radiotherapy (time point D). 12 months after the end of radiotherapy (time point E), 24 months after the end of radiotherapy (time point F), and 36 months after the end of radiotherapy (time point G). Each dimension of the questionnaire has at least 1 score, otherwise it is excluded. The questionnaires for all completed question groups are calculated based on actual scores, and the questionnaires for partially completed question groups are calculated based on average scores.

#### 2.3.3 Follow-up methods

Acute radiation injury was evaluated using the RTOG standard. The questionnaires at time points A and B were jointly completed by the doctor and the patient, and the other time points were performed by telephone follow-up and outpatient follow-up. Examines were performed every 3 months in the first 2 years, and every 6 months during the 3rd to 5th years. The examine items include pelvic magnetic resonance, abdominal ultrasound, chest X-ray, blood routine, liver function, kidney function, prostate specific antigen, serum testosterone, etc.

## 3 Statistical methods

Using SPSS13.0 (Verson1.0) software, clinical data is expressed in the form of mean ± standard deviation, and the dimensional score in the EPIC scale is expressed as mean, with time point A as the reference object, Mann–Whitney test is used to compare the difference between each time point and time point A; GraphPad Prism5 was used to draw the graph; Kaplan–Meier method was used to calculate IBF, PCSS, and OS, and Log-rank test of survival difference; Cox risk regression model was used for multivariate analysis of survival. *P* < .05 was considered statistically significant.

## 4 Result

### 4.1 General information

A total of 103 patients with prostate cancer met the inclusion criteria. Twelve patients with incomplete data were excluded. A total of 91 patients (88.3%) were included in the study, of which 52 (57.1%) received PODT and 39 (42.9%) received PMRT. The valid questionnaires at each follow-up point were comparable in general information such as age, baseline PSA level, number of pelvic lymph node metastases, and number of oligometastases in the 2 groups (Table 1).

**Table 1:** Comparison of characteristic data between PODT group and PMRT group (n, %).

Items	PODT (n = 52)	PMRT (n = 39)	*χ*^2^	*P*
Age			0.01	.91
≤65	17 (48.6)	12 (30.8)		
>65 and ≤75	35 (51.4)	26 (69.2)		
PSA (ng/ml)			0.04	.84
>20	3 (5.8)	2 (5.1)		
≤20	46 (94.2)	37 (94.9)		
Pelvic lymph node metastasis			0.31	.58
Y	13 (25.0)	8 (20.5)		
N	38 (75.0)	31 (79.5)		
Gleason scores			1.10	.58
≤6	5 (9.6)	2 (5.1)		
=7	6 (11.5)	3 (7.7)		
≥8	41 (78.9)	34 (87.1)		
No. of bone metastases			0.64	.89
1	8 (15.4)	6 (15.3)		
2	27 (51.9)	19 (48.7)		
3	14 (26.9)	10 (25.6)		
4	3 (5.8)	4 (10.4)		
ECOG scores			0.65	.72
0	17 (32.7)	16 (41.0)		
1	28 (53.8)	19 (48.7)		
2	7 (13.5)	4 (10.3)		

### 4.2 Comparison of OAR irradiation volume of PODT and PMRT tumor target areas

**Figure 1:**
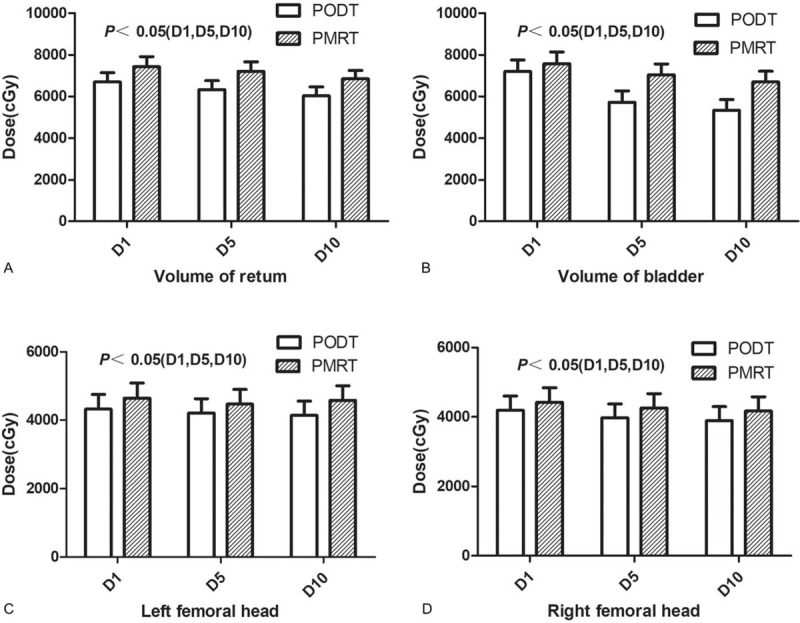
Comparison of OAR irradiation volume of tumor target area between 2 groups of patients (A is rectal irradiation dose, B is bladder irradiation dose, C is left femoral head irradiation dose, D is right femoral head irradiation dose).

The volume of PODT (*V*_podt_) is (264.52 ± 86.37) cm^3^ and the volume of PMRT (*V*_pmrt_) is (418.47 ± 63.64) cm^3^ (*P* < .05). The doses of D1, D5, and D10 in the rectum, bladder, left femoral head, and right femoral head of the PODT group were significantly lower than those of the PMRT (*P* < .05) (Fig. 1).

### 4.3 Comparison of quality of life between PODT and PMRT

**Figure 2:**
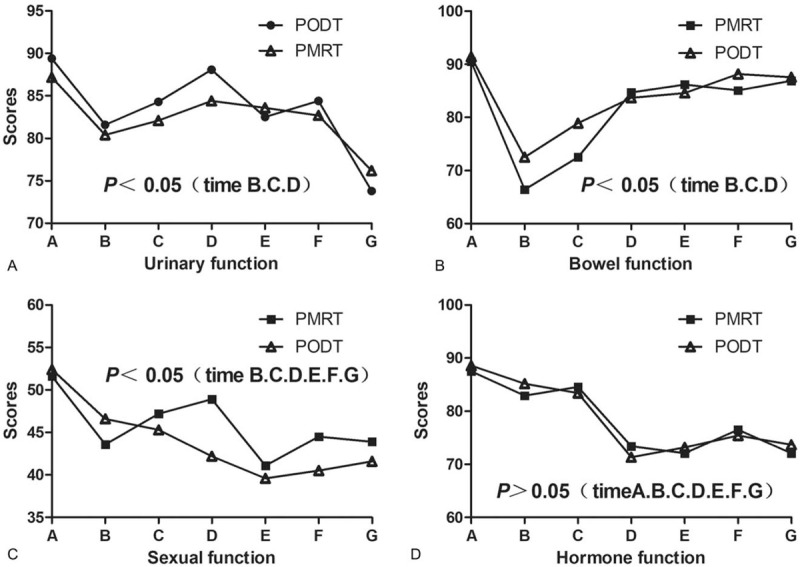
Comparison of EPIC scores between 2 groups of patients (a is urinary function score, b is intestinal function score, c is sexual function score, and d is hormone function score). Note: Time point A = baseline assessment; time point B = day of radiotherapy end; time point C = 3 mo after radiotherapy end; time point D = 6 mo after radiotherapy end; time point E = 12 mo after radiotherapy end; time point F = 24 mo after the end of radiotherapy; time point G = 36 mo after the end of radiotherapy.

The scores of urinary system function and intestinal system function in PMRT group were significantly higher than those of the PODT group on time B, C, and D (*P* < .05); there were no statistical differences at other follow-up time points (*P* > .05). The score of sexual function in the PODT group showed a continuous decrease during treatment, the differences were significantly difference on time B, C, D, E, F, and G; the scores of hormone function were no statistical difference between the groups (*P* > .05) (Fig. 2).

### 4.4 Comparison of follow-up between PODT and PMRT

**Figure 3:**
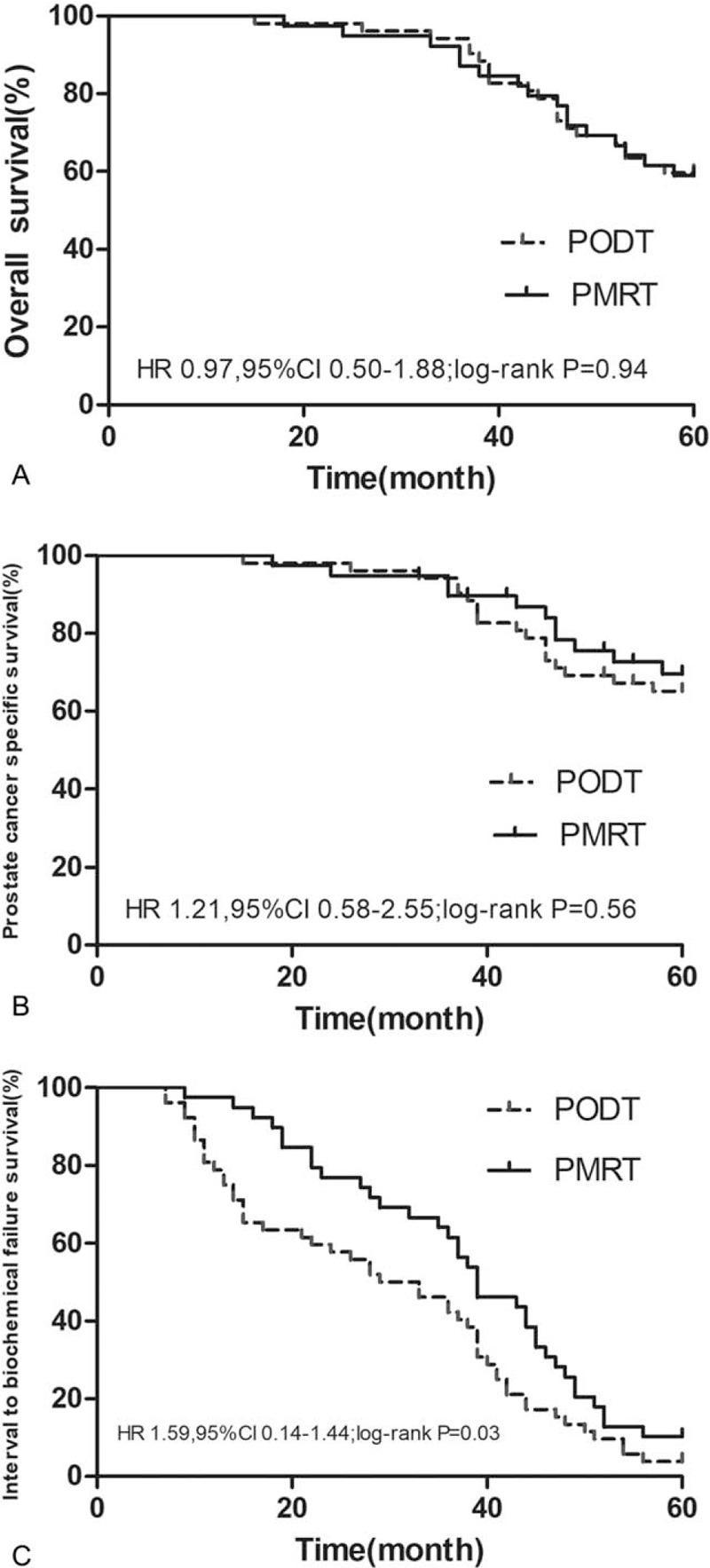
Survival analysis curves of 2 groups of patients (A is the OS survival curve; B is the PCSS survival curve; C is the IBF survival curve).

As of January 1, 2020, the 5-year OS of the PODT group was 59.6%, which was higher than the 58.9% of the PMRT group. There was no statistical difference between the groups (*P* = .94); the 5-year PSCC of the PODT group was 65.3%, which was lower than 71.79% of the PMRT group. There was no statistical difference between the groups (*P* = .56); the median IBF of the PODT group was 31 months and the median IBF of the PMRT group was 39 months. The comparison between the groups was statistically significant (*P* = .03) (Fig. 3).

### 4.5 Acute radiation injury

The incidences of leukopenia, thrombocytopenia, and anemia in the PMRT group were 82.1%, 66.7%, and 84.6%, which were significantly higher than the 57.7%, 48.1%, and 57.7% in the PORT group. The comparison between groups was statistical difference (*P* < .05). PMRT group showed 2 cases of 3 to 4 degree leukopenia, which returned to normal after treatment. The incidences of diarrhea and frequent urination in the PMRT group were 64.1% and 51.2%, which were higher than the 63.5% and 50% of the PODT group, there was no statistical significance between the groups (*P* > .05). The incidence of vomiting in the PMRT group was 48.7%, which was significantly higher than the 19.2% in the PODT group (*P* < .05) (Table 2).

**Table 2:** Comparison of acute radiation injury between PODT group and PMRT group (n, %).

	PODT group (n = 52)			PMRT group (n = 39)		
	Grade 1	Grade 2	Grades 3–4	Grade 1	Grade 2	Grades 3–4
Laboratory abnormalities						
White blood cells	27 (51.9)	3 (5.8)	–	26 (66.7)	4 (10.3)	2 (5.1)
Platelets	24 (46.2)	1 (1.9)	–	25 (64.1)	1 (2.6)	–
Hemoglobin	29 (55.8)	1 (1.9)	–	32 (82.1)	1 (2.6)	–
Clinical adverse events						
Diarrhea	32 (61.5)	1 (1.9)	–	24 (61.5)	2 (5.1)	–
Frequent urination	25 (48.1)	1 (1.9)	–	19 (48.7)	1 (2.6)	–
Vomiting	9 (17.3)	1 (1.9)	–	16 (41.0)	3 (7.7)	–

## 5 Discussion

Our previous studies have confirmed the advantage of the EPIC scale in evaluating acute radiation injury in prostate cancer patients undergoing radiation therapy.^[4]^ The treatment model and sequence of treatment of LBMP are the current research hotspots, including new endocrine therapy drugs (abiraterone, etc), docetaxel, etc, but the role of radiation therapy in LBMP is often ignored.^[5,6]^ To evaluate the efficacy of radiotherapy combined with endocrine therapy in patients with LBMP, compare the advantages and disadvantages of PODT and PMRT, and evaluate the efficacy, radiotherapy indicators, and quality of life and other objective indicators, can provide visual data reference for the treatment of patients with LBMP.

Target volume delineation is an important part of radiotherapy. The evaluation of target volume and OAR is the main reference index for evaluating radiotherapy.^[7]^ Although proton therapy has advantages in OAR dose reduction for prostate cancer, existing research cannot prove that proton therapy can replace intensity modulated radiation therapy (IMRT).^[8]^ IMRT is still one of the mainstream technologies for prostate cancer radiotherapy. In our study, there is a great difference in the target volume demarcation between the 2 groups. The main reason is that PMRT increases the scope of metastases. The volume of irradiated tumor, the dose of D1, D5, and D10 of OAR significantly increased, and the increase of tumor volume is a risk factor of prognosis.^[9]^*V*_pmrt_ is higher than *V*_podt_, but there is no difference in long-term survival. Whether tumor radiotherapy volume can be used as a prognostic indicator is not clear. The ROC curve may be used to further classify patients with the best tumor volume for radiation therapy, but further confirmation is needed. How to reduce the dose of the rectum and other important organs that are at risk is the key to increasing the dose of prostate lesions. The use of hydrogel to inject connective tissue between the prostate and the rectum can effectively reduce rectal volume.^[10]^ Due to the limitations of the study conditions, none of our subjects used similar techniques, so the rectal volume of the PODT group and the PMRT group increased significantly, which was also significantly higher than the data reported in other studies.^[11]^ It may be related to the fact that external radiation increases the target area of radiation therapy and increases the risk of rectal injury.^[12]^ The outline of the PMRT group included the pelvic lymph node area, which greatly increased the irradiation dose of the bladder and bilateral femoral heads. The degree of bladder filling directly affected the internal movement range of the prostate.^[13]^ The dose of bladder irradiation in both the PMRT group and the PODT group was higher than in previous studies, which may be related to the heterogeneity of the study population. In addition, exercise of bladder filling capacity before positioning may reduce the radiation dose of the bladder.^[14]^

The quality of life of patients with metastatic prostate cancer often determines the choice of treatment strategy.^[15]^ At 5 years of follow-up, the urinary and intestinal function scores of the PODT group were higher than those of the PMRT group within 6 months of the end of radiotherapy, and decreased significantly at the end of the radiotherapy, which was closely related to the high dose of radiation to the organs at risk. Acute urinary and intestinal reactions above 2 degrees in the study group were significantly higher than 1.4% and 9.3% reported by Pasquier et al,^[16]^ the latter uses external radiation combined with stereotactic radiation therapy. We used external radiation throughout the study. The main research object was M1 patients. Some patients also received radiotherapy for metastatic lesions, which increased the risk of urinary and intestinal reactions. When the EPIC sexual function score is between 40 and 60, 28% of patients with prostate cancer after surgery have normal erectile function.^[17]^ Our research subjects are mainly elderly men, and they all receive long-term androgen deprivation therapy. Therefore, the overall decline in sexual and endocrine functions during follow-up period. There was no significant correlation between testosterone level and prostate cancer, but it was closely related to bone density and sexual function.^[18]^ The sexual function of the PMRT group improved significantly after 3 months of radiotherapy, which may be related to better control of bone metastases.

Our previous studies have found that patients with locally advanced prostate cancer who have undergone radiation therapy have a smaller exposure range and a lower incidence of bone marrow suppression.^[19]^ Decreased white blood cells, platelets, and hemoglobin are the main hematological toxic reactions during prostate cancer radiotherapy, and the active bone marrow of the pelvis plays an important role in the hematopoietic function of the body.^[20,21]^ This may be the main reason for the hematological toxicity of PMRT group (increasing bilateral femoral head and bone metastases) to be higher than that of PODT group. Acute injuries caused by radiotherapy, including diarrhea and vomiting, can be assessed by the patients themselves, which can minimize the false negative rate caused by doctors’ evaluation.^[22]^ In our study, EPIC and RTOG assessments can be used to more objectively evaluate acute radiation injury. The dose of rectal and bladder irradiation was increased in the group of PMRT, resulting in higher incidence of diarrhea, urinary frequency, and other symptoms. Whether the differences in prostate movement variability in the PMRT group and the PODT group during radiotherapy can cause differences in acute radiotherapy injury^[23]^ needs further confirmation. Radiotherapy-induced vomiting is one of the main factors affecting the quality of life.^[24]^ The incidence of vomiting in the PMRT group was higher than that in the PODT group, but no vomiting of 3 degrees or more occurred in the entire group. Both radiotherapy modes are tolerable for patients.

Sandler et al^[25]^ conducted a retrospective study on the role of pelvic prophylactic radiation in the treatment of prostate cancer radiotherapy, and confirmed that increasing pelvic radiation cannot improve OS and PCSS. Compared with our study, there are differences between the 2 in terms of research objects and radiotherapy methods, but indirectly confirm that the increase in target volume does not improve long-term survival. It also shows that methods including endocrine therapy have an important role in improving the overall survival of prostate cancer. Bowden et al used stereotactic radiotherapy technology, based on local prostate treatment, the effect of irradiation between 4 to 5 and 1 to 2 metastases did not show a difference.^[26]^

Our research found that PMRT has a better advantage in improving IBF. The tumor biological effects of different radiation treatment techniques and radiation doses still differ, but long-term survival is largely the same regardless of the mode of radiotherapy. In addition, the importance of the pelvic lymph nodes has often been ignored in previous studies of LBMP.^[27]^

The PMRT group in our study included lymph nodes in the irradiation field, but did not reflect the survival advantage, and a larger sample size is needed to confirm whether further stratified analysis will yield positive results. The results of a retrospective cohort study found that the 5-year OS and PCSS of radiotherapy combined or partial metastases based on brachytherapy were 64.4% and 87.9%,^[28]^ respectively, which were significantly higher than those in the PODT and PMRT groups. As a retrospective study, due to the restriction of the overall medical level in China, the use rate of most newly developed drugs is still lower than that in Europe and the United States, which may be the main factor contributing to the survival difference.

As a retrospective study, there is no valid data to confirm whether PMRT can increase the radiation dose to other OAR organs, such as the lungs, heart, etc. Due to the limitation of the research conditions, the long-term radiation injury caused by PMRT and PODT was not followed up. To sum up, on the basis of endocrine therapy, prostate lesions combined with radiotherapy for metastatic lesions in LBMP patients may lead to greater urinary and digestive system toxicity, although increased IBF, but does not improve long-term survival.

## Acknowledgments

We thank all the survey respondents who participated in the study and survey field staff who did the interviews. We thank Jian-Hua Wu and Na Li for promoting the survey and Yong-Hai Peng for many helpful suggestions.

## Author contributions

Luo HC, Fu ZC, and Liao SG contributed to the study concept. Chen ZH contributed to the study design. Luo HC and Fu ZC wrote the manuscript. Wang FM, Wang XP, and YQ reviewed and edited the manuscript. Lin GS in charge of collecting data. All authors read and approved the final manuscript.

**Conceptualization:** Huachun Luo, Zhi-Chao Fu, Zhong-Hua Chen, Shao-Guang Liao.

**Data curation:** Lv-Juan Cai, Qin Yin, Guishan Lin, Feng-Mei Wang.

**Funding acquisition:** Zhong-Hua Chen.

**Methodology:** Guishan Lin.

**Visualization:** Xin-Peng Wang, Qin Yin, Feng-Mei Wang.

**Writing – original draft:** Huachun Luo.

**Writing – review & editing:** Huachun Luo, Zhi-Chao Fu, Shao-Guang Liao, Feng-Mei Wang.
